# A Novel Function of Novobiocin: Disrupting the Interaction of HIF 1α and p300/CBP through Direct Binding to the HIF1α C-Terminal Activation Domain

**DOI:** 10.1371/journal.pone.0062014

**Published:** 2013-05-06

**Authors:** Donglu Wu, Rui Zhang, Rui Zhao, Guang Chen, Yong Cai, Jingji Jin

**Affiliations:** 1 College of Life Science, Jilin University, Changchun, Jilin, China; 2 Graduate School of Jilin University, Changchun, Jilin, China; 3 The First Clinical Hospital, Jilin University, Changchun, Jilin, China; 4 National Engineering Laboratory for AIDS Vaccine, College of Life Science, Jilin University, Changchun, Jilin, China; Vanderbilt University, United States of America

## Abstract

Hypoxia-inducible factor 1α (HIF1α) is an important cellular survival protein under hypoxic conditions, regulating the cellular response to low oxygen tension via recruitment of a transcriptional co-activator, p300/CBP. p300/CBP induces expression of multiple genes involved in cell survival, proliferation, angiogenesis, and tumor development. Thus, a strategy to inhibit hypoxic responses in tumors may be to target the protein-protein interaction between HIF1α and p300/CBP. Here, we document, for the first time, that the aminocoumarin antibiotic, novobiocin, directly blocks the protein-protein interaction between the HIF1α C-terminal activation domain (CTAD) and the cysteine-histidine rich (CH1) region of p300/CBP. Also, novobiocin down-regulated HIF1α-controlled gene expression, specifically CA9, which is related to tumorigenesis. In a monolayer cell culture, novobiocin inhibited cell proliferation and colony formation in the MCF-7 human breast adenocarcinoma cell line and the A549 human lung cancer cell line. Rescue experiments revealed that the recombinant CTAD fragment of HIF1α partially reversed novobiocin’s inhibitory effects on cell proliferation and colony formation in MCF-7 cells. These findings suggest a novel mechanism of action for novobiocin which has the potential for innovative therapeutic use in tumor treatment.

## Introduction

Hypoxia is critical for tumors because hypoxic conditions render a more aggressive tumor phenotype with increased invasiveness and proliferation, thereby increasing metastatic growth and decreasing patient survival [1]. Hypoxia inducible factor 1α (HIF1α), a member of the basic helix-loop-helix (bHLH)-PAS (period circadian protein, aryl hydrocarbon-receptor nuclear translocator, single-minded protein) family, is necessary under hypoxic conditions that frequently occur in several carcinomas and their metastases [2]. Under normoxic conditions, HIF1α is hydroxylated in the oxygen-dependent degradation domain (ODD domain) by HIF prolyl-hydroxylases (PHD). This permits recognition and ubiquitination by VHL E3 ubiquitin ligase, leading to rapid proteasomal degradation [3]. However, HIF1α is not hydroxylated under hypoxic conditions, and cellular levels increase because the VHL ubiquitin ligase complex cannot bind HIF1α to promote its ubiquitination and degradation. As a result, accumulated HIF1α translocates to the nucleus, where it dimerises with HIF1β. HIF1α recruits transcriptional co-activators such as p300/CBP (p300/CREB-binding protein) and binds to the hypoxia-response element (HRE). Such binding activates hypoxically regulated genes, such as vascular endothelial growth factor (VEGF), glucose transporter 1 (GLU-1) and carbonic hydrase IX (CA9) [4]–[7]. Hypoxia is a hallmark of solid tumors because rapidly dividing tumor cells receive insufficient oxygen from the vascular system [8], an event that has been reported to activate the HIF pathway.

The interaction between the HIF1α C-terminal activation domain (CTAD) and the cysteine-histidine rich (CH1) region of p300/CBP transcriptional co-activators is critical for HIF1α transactivation. Blocking this interaction reduces transcriptional activation of HIF1α [9], [10]. Chetomin, a dimeric epipolythiodiketopiperazine (ETP), is a metabolite of several species of *Chaetomium* fungi. As an inhibitor of the HIF pathway, chetomin blocks the interaction of HIF1α and HIF2α with transcriptional co-activators p300/CBP. This attenuates hypoxia-inducible transcription, which consequently decreases tumor growth and reduces downstream VEGF gene expression [10]–[12]. Kushal *et al.* designed and synthesized the ETP3 derivative of ETP and reported that ETP3 binds to the target p300/CBP CH1 domain with micromolar affinity and disrupts the formation of the HIF1α CTAD/p300/CBP complex *in vitro*. In a cell-based assay, ETP3 disrupted the structure and function of the HIF1α CTAD/p300/CBP CH1 complex in a dose-dependent manner, rapidly down-regulating hypoxia-inducible expression of the VEGF gene in MDA-MB-231 and MCF-7 cells [11]. Therefore, a strategy to inhibit the hypoxic tumor response may be to target the binding and subsequent interaction of HIF1α CTAD and p300/CBP CH1 domains.

Supporting such a strategy, various research groups have identified small compounds that inhibit the HIF-1 pathway via different mechanisms: affecting HIF1α synthesis and degradation, HIF1α-HIF1β dimerization, DNA binding, and altering other proteins important for transcriptional activities [8]. For instance, geldanamycin (GA), a benzoquinone ansamycin antibiotic, is a naturally occurring inhibitor of heat shock protein (Hsp90), which regulates the transcription activity of HIF-1α. Experimental data show that GA inhibits angiogenesis and invasion mediated by HIF-1α in prostate cancer cells (DU-145 cells) [13]. Whereas amphotericin B (Am B), a polyene antifungal drug, specifically repressed the CTAD of HIF-1α, and this repression required Asn 803, a target site of the factor-inhibiting HIF-1 (FIH). Furthermore, Am B stimulates CTAD-FIH interaction and inhibits p300 recruitment [14]. Recent research indicates that hypericin, a structurally related anthraquinone, can decrease excessive angiogenesis by degrading HIF1α in tumor cells via a unique hypoxia- and proteasome-independent mechanism [15]. The aminocoumarin antibiotic novobiocin, a potent inhibitor of bacterial DNA gyrase, can also interact with Hsp90 and disrupt its chaperone activity in a manner similar to GA and radicicol [16]. In addition, novobiocin and its analogues have been shown to have anti-cancer effects through Hsp90 inhibition [17], [18]. However, the mechanism by which novobiocin inhibits tumor growth is unclear. Here, we report, for the first time, that novobiocin can directly disrupt the HIF1α CTAD/p300 CH1 complex. We also report that the inhibitory function of novobiocin on the HIF1α/p300 complex might be important in cell growth and colony formation within human lung cancer cells (A549) and breast cancer cells (MCF-7). These findings suggest a novel molecular anti-tumor mechanism of novobiocin.

## Materials and Methods

### Materials

Anti-FLAG (M2) agarose, anti-FLAG (M2) monoclonal antibody, anti-FLAG peptide, novobiocin (N-1628), cisplatin (479306) and galdanamycin (G3381) were purchased from Sigma Aldrich (St. Louis, MO). Anti-HIF1α H206 (Cat# sc-10790) and anti-p300 N-15 (Cat# sc-584) rabbit polyclonal antibodies were obtained from Santa Cruz Biotechnology (Santa Cruz, CA). Anti-GST monoclonal antibody (Cat# MAB1132) was from the Abnova Corporation (Taiwan). Anti-HPC4 antibody was from Roche Applied Science (Germany).

### HeLa Nuclear Extract Preparation and Protein Affinity Purification

HeLa S3 cells were grown in spinner culture in Joklik medium with 5% calf serum. Nuclear extract was prepared according to the method of Dignam *et al.* [19]. N-terminally His-GST-tagged human HIF1α 425–826, HIF1α 776–826, and p300 CH1 were expressed from pET41 in BL21 (DE3) Codon Plus *Escherichia coli.* bacterial cells. N-terminally His-tagged GST was expressed from pET19b in BL21 (DE3) Codon Plus *Escherichia coli* bacterial cells. Expressed proteins were his-affinity purified by incubating Ni-NTA agarose (Qiagen, Valencia, CA) at 4°C for 4 hours. cDNAs encoding wild-type full-length HIF1α containing N-terminal His epitope tags and the CH1 domain of p300 containing N-terminal Flag epitope tags were subcloned into pBacPAK8. Recombinant baculoviruses were generated with the BacPAK expression system (Clontech Laboratories, Inc). Sf21 insect cells were infected and cultured at 27°C in Sf-900 II SFM (Invitrogen). 48 hours after infection, cells were collected and lysed in ice-cold buffer containing 50 mM Hepes-NaOH (pH 7.9), 0.5 M NaCl, 5 mM MgCl_2_, 0.2% Triton-X-100, 10% (vol/vol) glycerol. Lysates were centrifuged 100,000×g for 30 minutes at 4°C. His-affinity purifications were performed by incubating Ni-NTA agarose (Qiagen) at 4°C for 4 hours with cell lysates adjusted to His-binding buffer. Beads were then washed with 50 ml binding buffer with 20 mM imidazole 4 times. Proteins were eluted with 300 mM imidazole in 40 mM HEPES-KOH at pH7.9, 150 mM NaCl, 10% glycerol and 0.05% Triton-X-100.

### Novobiocin Immobilized Beads Preparation

Novobiocin-sepharose was prepared according to the method of Monica *et al.* [16]. Briefly, 1.5 g epoxy-activated sepharose 6B gel (Sigma) was mixed with 200 mg of novobiocin (Sigma) in 5 ml of coupling buffer (0.3 M sodium carbonate, pH 9.5) and incubated at 37°C with gentle rotation for 18–20 hours. Excess ligand was washed away and the remaining epoxy-active groups were blocked with 1 M ethanolamine in coupling buffer for 12 hours at 30°C with gentle shaking. The gel was then washed sequentially with coupling buffer, 0.5 M NaCl in coupling buffer, distilled water, 0.5 M NaCl in sodium acetate (pH 4) buffer, and then washed again in distilled water. Finally, the gel was equilibrated in 25 mM Hepes (pH 8) containing 1 mM EDTA, 10% ethylene glycol, 200 mM KCl and kept at 4°C, protected from light until needed.

### Luciferase Reporter Assay

293T cells were cotransfected with 1 µg of pG5-Luc (Promega), which encodes firefly luciferase driven by GAL4 sites upstream of the AdML core promoter; 100 ng of the control plasmid pRL-tk (Promega), which encodes *Renilla* luciferase under control of the thymidine kinase promoter; and varying amounts of effector plasmid expressing GAL4-HIF1α DBD or GAL4-HIF1α CTAD, using FuGene 6 reagent (Roche). Total effector plasmid in each transfection was adjusted to 1 µg with empty vector. After 48 hours, GAL4-HIF1α transactivation activity was determined by measuring firefly and *Renilla* luciferase activities using the Dual-Luciferase Reporter assay kit (Promega) and by normalizing firefly to *Renilla* luciferase.

### Cell Culture and Maintenance

Human HEK293T, lung carcinoma type II epithelium–like A549 (ATCC#: CCL-185), human breast adenocarcinoma MCF-7 (ATCC#: HTB-22™) and human breast cancer BT474 (ATCC#: HTB-20™) cells were originally obtained from the American Type Culture Collection (ATCC, Manassas, VA). All cells were cultured in Dulbecco’s Modified Eagle’s Medium (DMEM, Sigma) with 5% glucose and 10% fetal bovine serum (FBS), 100 U/mL penicillin, 100 mg/mL streptomycin in 10 cm dishes at 37°C in a humidified atmosphere of 5% CO_2_. Whole-cell extracts were prepared from cells from 1 well of a 6-well plate by adding 4×SDS sample buffer.

### Reverse Transcription PCR (RT-PCR)

Cells were harvested from 1 well of a 6-well plate and total RNA was isolated using TRIzol® LS Reagent (Invitrogen). 1 µg of RNA from each sample was used as a template to produce cDNA with PrimeScript 1st Strand cDNA Synthesis Kit (TAKARA). HIF1α, VEGF, CAlX (CA 9), HIF2α, HIF1β, FLJ20436, KIAA1267, Akt1, mTOR or GAPDH mRNA levels were analyzed by polymerase chain reaction (PCR) with a C1000™ Thermal Cycler (Bio-Rad). All PCR reactions were finished as follows: an initial denaturation step was performed at 95°C for 3 minutes, followed by 35 cycles of denaturation at 95°C for 30 seconds, annealing at 60°C for 30 seconds, and extension at 72°C for 30 seconds. The primer sets used for PCR were as follows: HIF1α, 5′-GCACAGGCCACATTCACG-3′ (forward) and 5′-TGAAGATTCAACCGGTTTAA GGA-3′ (reverse), yielding a 520-bp product; VEGF, 5′-GAACTTTCTGCTGTCTTGGGTGCAT-3′ (forward) and 5′-GGTCTGCATTCACATTTGTTGTGCTG-3′ (reverse), yielding a 392-bp product; CA9, 5′-GCAGGAGGATTCCCCCTTG-3 (forward) and 5′-GGAGCCTCAACAGTAGGTAGAT-3′ (reverse), yielding a 185-bp product; HIF2α, 5′-CAACCTCAAGTCAGCCACCT-3′ (forward) and 5′-TGCTGGATTGGTTCACACA TG-3′ (reverse), yielding a 143-bp product; HIF1β, 5′-CAGGACCAGAACACAGC AA-3′ (forward), and 5′-GCAGAAGCTGATGGCTGG-3′ (reverse), yielding a 207-bp product; GAPDH, 5′-ATCACTGCCACCCAGAAGAC-3′ (forward), and 5′-ATGAGGT CCACCACCCTGTT-3′ (reverse), yielding a 460-bp product.

### Soft-agar Colony Formation Assay

The anchorage-independent growth of A549 and MCF-7 cells with or without 200 µM novobiocin was estimated by a soft-agar colony formation assay as described [20]. Single-cell suspensions of 2.5×10^3^ cells were plated per 6-well plates in 1 mL of DMEM containing 10% fetal bovine serum (FBS) and 0.35% agar on a layer of 1 mL of the same medium containing 0.5% agar. Three weeks after plating, the colonies were stained with 0.005% crystal violet. Photographs of the stained colonies were taken and the number of foci >100 µm was counted.

### Cell Growth Assay

The proliferation of A549 and MCF-7 cells were evaluated by the cell-counting method. A549 and MCF-7 cells were seeded in 12-well plates (∼2×10^4^ cells/well) and allowed to grow for different periods. The growth rate was determined by the cell number, counted with a hemacytometer in triplicate every day of the culture, up to the seventh day.

### Statistical Analysis

Statistical analysis was completed with SPSS 17.0 (SPSS, Inc., Chicago IL). Statistical comparisons were analyzed using the student’s *t*-test. Values of *P*<0.05 were considered to be statistically significant.

## Results

### Novobiocin can Disrupt the HIF1α CTAD/p300 CH1 Complex

**Figure 1:**
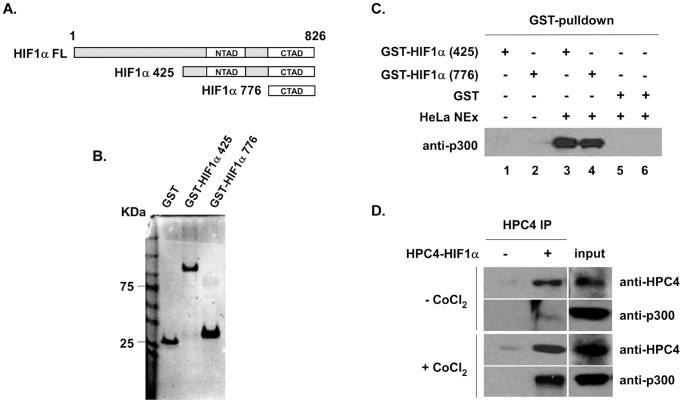
HIF1αCTAD interacts with p300. **A.** Deletion structure of HIF1α. The schematic diagram indicates their length and location compared with full-length HIF1α. **B.** Fragments of HIF1α were cloned and expressed as His-GST fusion proteins and were analyzed by SDS-PAGE (4–20% gradient gel, BioRad). Proteins were visualized by Coomassie Blue staining. **C.** Deletion HIF1α 425 and HIF1α 776 bind to endogenous p300: ∼10 µg of bacterially expressed and GST purified HIF1α 425 and HIF1α 776 (CTAD) were mixed with 100 µl of HeLa nuclear extract and 15 µl of glutathione-sepharose 4 Fast Flow beads (Amersham Biosciences) in binding buffer containing 40 mM Tris-HCl (pH 7.4), 150 mM NaCl, and 0.2% (v/v) Triton X-100. Protein mixtures were incubated for 4 hours at 4°C and bound proteins were eluted with elution buffer containing 25 µl of 30 mM glutathione, 40 mM Tris-HCl (pH 7.5), 10% (v/v) glycerol, and 150 mM NaCl. Pulled-down p300 was confirmed by Western blotting with anti-p300 antibody. **D.** Overexpressed full length (FL) HIF1α interacts with endogenous p300 in the presence or absence of CoCl_2_. HeLa cells were transiently transfected with 1 µg of HPC4-HIF1α. Hypoxia conditions were induced by adding CoCl_2_ (Sigma, 150 µM). Proteasome inhibitor MG132 (50 µM) was added to block protein degradation and assess the total amount of HIF1α 3 hours before harvest. Then, 72 hours after HPC4-HIF1α transfection, cells were harvested and lysed for immunoblot.

HIF1α CTAD is known to interact with p300 [21], [22]; thus, to confirm that HIF1α directly binds to p300, we designed different fragment lengths of HIF1α and introduced these fragments into a pET41 vector (Fig. 1A). GST fusion HIF1α 425 and HIF1α 776 were expressed in *E. coli* and were purified through glutathione-sepharose chromatography (Fig. 1B). A GST pull-down assay was then performed using GST-purified HIF1α 425 and HIF1α 776. Fig. 1C shows that both GST-HIF1α 425 and GST-HIF1α 776 (HIF1α CTAD) pulled-down endogenous full-length (FL) p300 from HeLa nuclear extracts (lanes 3 and 4) compared to GST (lanes 1 and 2). In addition, overexpressed HPC4-HIF1α immunoprecipitated endogenous p300 in HeLa cells (Fig. 1D).

**Figure 2:**
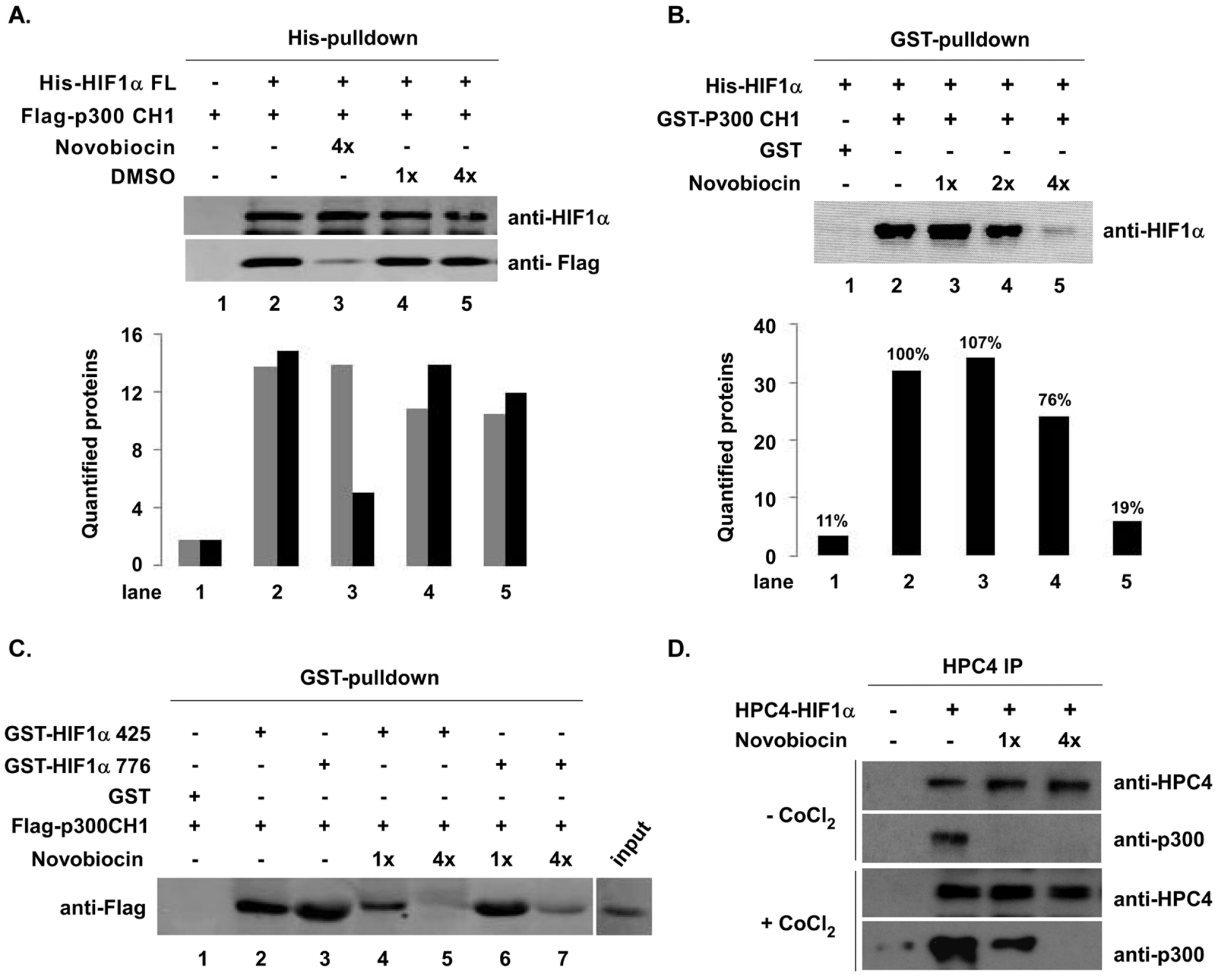
Novobiocin directly disrupts the HIF1αCTAD/p300 CH1 complex. **A.** Novobiocin inhibits His-HIF1α FL protein pulling down p300 CH1. Insect cell expressed and purified His-HIF1α FL (∼5 µg), Flag-p300 CH1, 400 µM of antibiotic novobiocin or DMSO (control), and 15 µl of Ni^2+^-agarose were mixed in binding buffer containing 40 mM Hepes-NaOH (pH 7.9), 250 mM NaCl, 10% glycerol, and 40 mM imidazol, and incubated at 4°C for 2 hours. Bound proteins were eluted with 20 µl of elution buffer containing 300 mM imidazol and were then confirmed by Western blotting with anti-HIF1α and anti-Flag antibodies (upper panel). Western blot images were quantified using Quantity One software (BioRad) (lower panel). **B.** Novobiocin inhibited GST-p300 CH1 protein pulling down the HIF1α FL. GST-p300 CH1 protein (∼10 µg GST only as a negative control) was mixed with His-HIF1α FL, 15 µl of glutathione sepharose 4 Fast Flow beads, and novobiocin (100–400 µM). Bound proteins were then detected by Western blotting with anti-HIF1α antibody. Quantified HIF1α is depicted in the graph (lower panel). *Percentages* given above *each bar* represent bound HIF1α protein compared to lane 2. **C.** Novobiocin directly disrupts the HIF1α CTAD/p300 CH1 complex. The GST pull-down assay was performed by mixing (as indicated in the figure) ∼10 µg GST fusion proteins, 5 µg Flag-p300 CH1, and 100 or 400 µM novobiocin. Pulled-down p300 CH1 was detected by Western blotting with anti-Flag antibody. **D.** Novobiocin inhibited the interaction between overexpressed HIF1α FL and endogenous p300 in the presence or absence of CoCl_2_. HPC4-HIF1α FL was overexpressed in HeLa cells in the presence or absence of novobiocin (100 or 400 µM). Hypoxic conditions were induced by the addition of CoCl_2_ (150 µM) for 8 hours. Then, 50 µM MG132 was added 3 hours before harvest. At 72 hours after HPC4-HIF1α transfection, cells were harvested and an HPC4 immunoprecipitation assay was performed by mixing 250 µl whole-cell lysate, 2 µg HPC4 antibody, and 15 µl of glutathione sepharose. Bound p300 was then detected by Western blotting with anti-p300 antibody.

To investigate whether novobiocin affected the interaction of HIF1α/p300, we examined the interaction of novobiocin with HIF1α FL and p300. Previous reports [22], [23] suggest that the p300 CH1 domain is the HIF1α-interacting region. Thus, we generated His-tagged HIF1α FL and Flag-tagged p300 CH1 in insect cells and purified proteins through Ni^2+^ chromatography. Purified His-HIF1α FL and Flag-p300 CH1 were applied to a His pull-down assay in the presence of novobocin or DMSO (control). As shown in Fig. 2A, His-HIF1α FL pulled-down Flag-p300 CH1 proteins (lane 2), and this protein-protein binding activity was significantly inhibited by novobiocin (lane 3), but not by DMSO (lanes 4 and 5). A reverse pull-down assay produced similar results, revealing that GST-p300 CH1 pulled-down His-HIF1α FL (Fig. 2B, lane 2), and this protein-protein binding activity was dose-dependently inhibited by novobiocin (Fig. 2B, lanes 3–5). Quantified HIF1α protein levels (Quantity One software) was reduced to 76% and 19%, respectively, compared to lane 2. To describe novobiocin’s inhibitory activity on the HIF1α/p300 complex in more detail, we carried out a GST pull-down assay with GST-HIF1α 425 and GST-HIF1α 776 as indicated in Fig. 2C. Either GST-HIF1α 425 or GST-HIF1α 776 pulled-down recombinant p300 CH1 (Fig. 2C, lanes 2 and 3) compared to GST (Fig. 2C, lane 1). Novobiocin blocked the interaction of either HIF1α 425 or HIF1α 776 with recombinant p300 CH1 (Fig. 2C, lanes 4–7). The interaction between overexpressed HPC4-HIF1α and endogenous p300 in the presence or absence of CoCl_2_ in HeLa cells was also inhibited by novobiocin (100–400 µM). These experiments suggest that novobiocin directly disrupts the HIF1α CTAD interaction with recombinant p300 CH1 and endogenous p300.

### Novobiocin Specifically Inhibits the Interaction between HIF1α CTAD and p300 CH1

**Figure 3:**
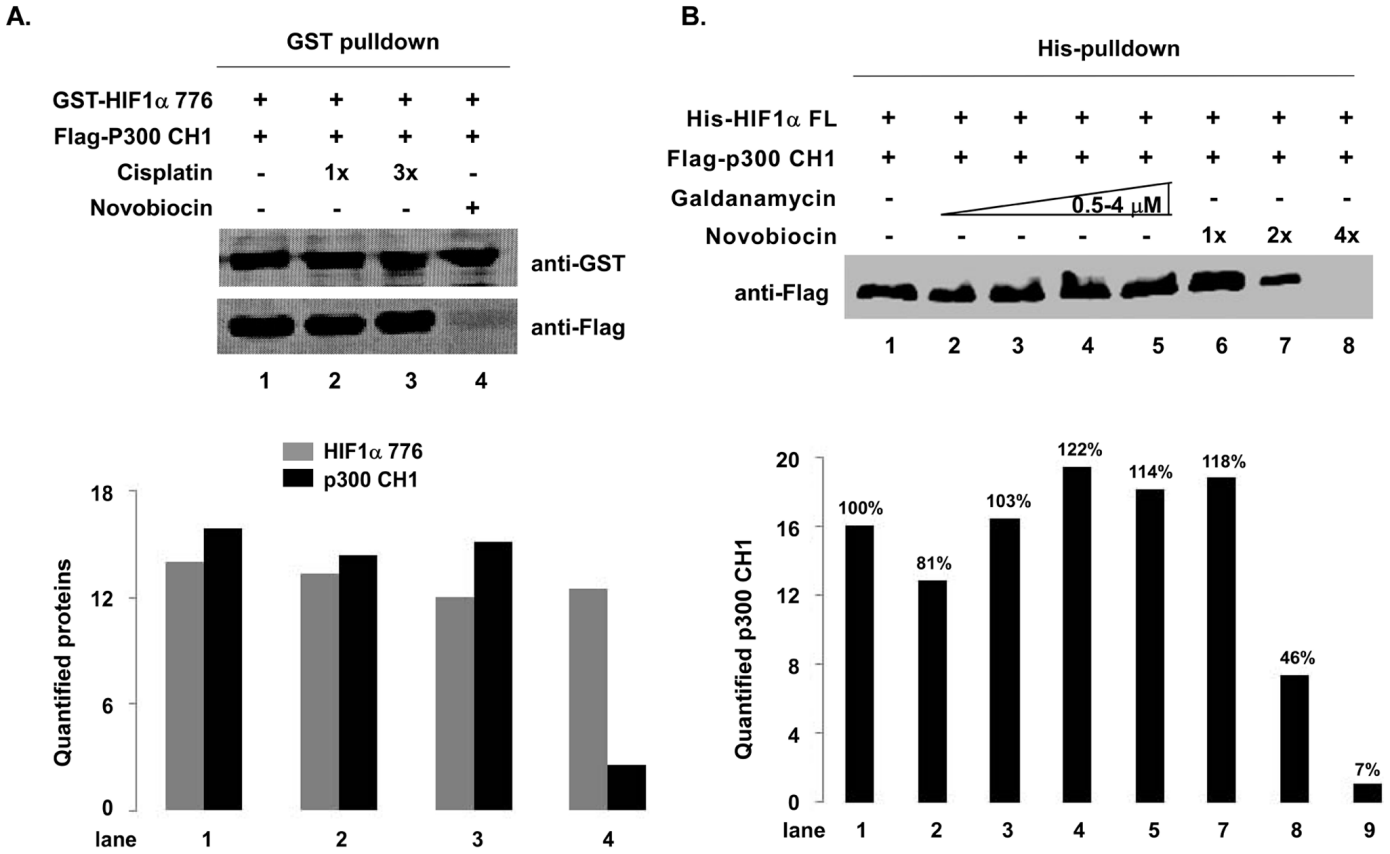
Novobiocin specifically inhibits the interaction between HIF1αCTAD and p300 CH1. **A.** Cisplatin did not disrupt the HIF1α CTAD/p300 CH1 complex. A GST pull-down assay was performed by mixing GST fusion HIF1α CTAD, Flag-p300 CH1, and novobiocin (300 µM) or cisplatin (10–30 µM). HIF1α CTAD and p300 CH1 were detected by Western blotting with anti-GST and anti-Flag antibodies (upper panel). Quantified proteins are depicted (lower panel). **B.** Galdanamycin, an inhibitor of Hsp90, does not affect the interaction between HIF1α and p300 CH1. His pull-down was carried out by mixing purified proteins (combinations indicated in the figure). Bound p300 CH1 protein was detected by Western blotting with anti-Flag antibody. Western blot images were quantified using Quantity One software (lower panel). P*ercentages* given above *each bar* represents the bound p300 CH1 protein compared to lane 1.

To learn whether the inhibition of novobiocin on HIF1α CTAD/p300 CH1 was specific, we investigated other known antibiotics or chemotherapeutic agents with similar mechanisms of action. The chemotherapeutic cisplatin (cis-diamminedichloro-platinum [II]. CDDP) binds to and crosslinks DNA, ultimately triggering apoptosis [23]–[25]. In our experiments, unlike novobiocin, cisplatin did not affect the interaction between HIF1α CTAD and p300 CH1 in the 10–30 µM range (Fig. 3A, lanes 2 or 3, compared to lane 1). In addition, although both novobiocin and galdanamycin (as an Hsp90 inhibitor) can bind to the C-terminal ATP binding site and lead to the degradation of Hsp90 client proteins [16], galdanamycin does not disrupt HIF1α/p300 CH1 complex interaction in the 0.5–4 µM range (Fig. 3B), suggesting different mechanisms of action for novobiocin and geldanamycin.

### Novobiocin Directly Binds to the HIF1α CTAD

**Figure 4:**
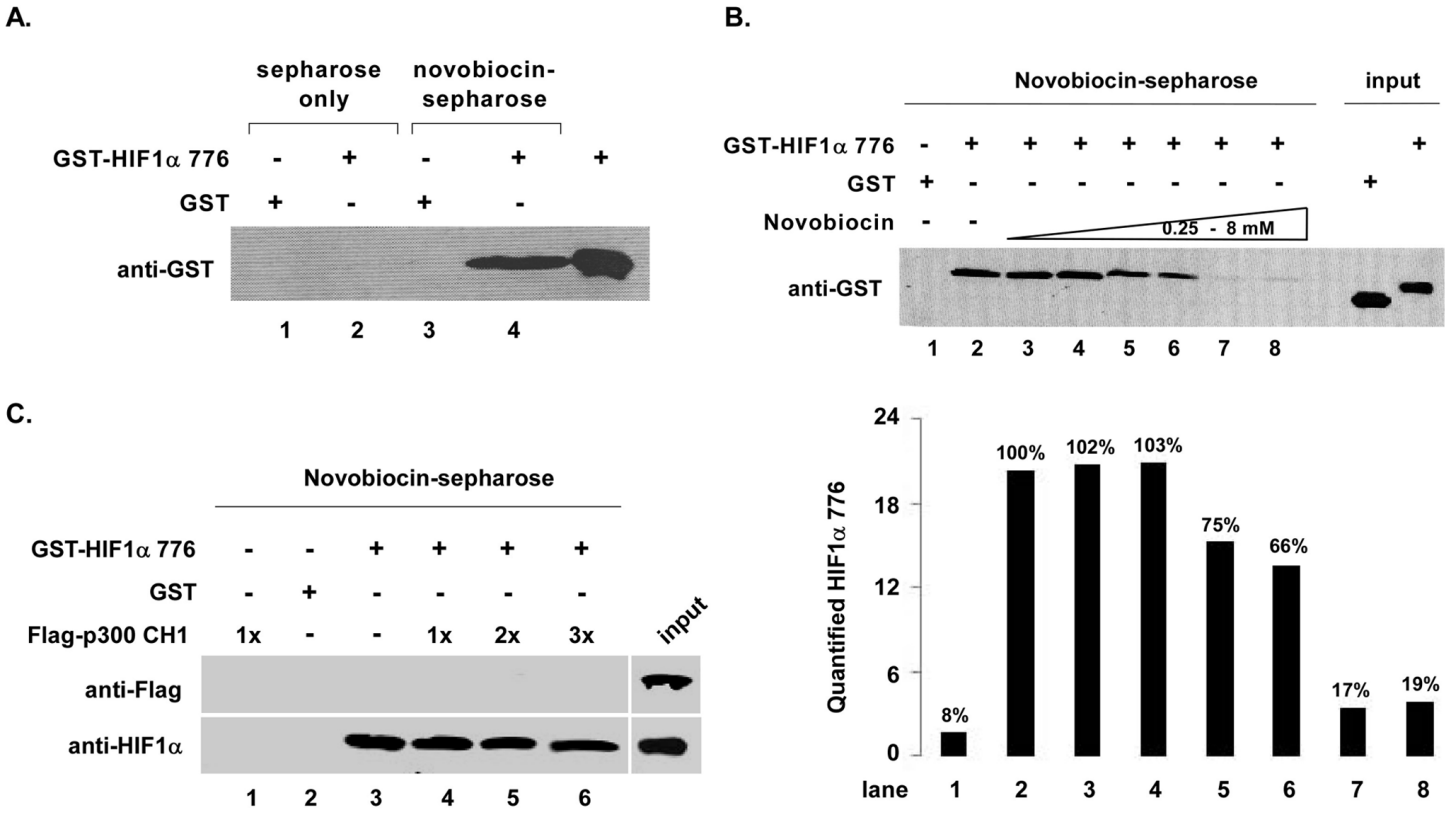
Novobiocin directly binds to HIF1α CTAD. **A.** Novobiocin-sepharose pulled down HIF1α CTAD. Immobilized novobiocin-sepharose was prepared as described under §Experimental Procedures. GST fusion HIF1α CTAD (∼5 µg) was mixed with novobiocin-sepharose or novobiocin-free sepharose in a buffer containing 40 mM Tris-HCl (pH 7.4), 1% NP-40, 2 mM EDTA, 100 mM NaCl, and 1 mM sodium orthovanadate, and a protease inhibitor cocktail (Sigma). Protein mixtures were incubated at 4°C for 2 hours, and the beads were washed with ice-cold buffer. Bound proteins were eluted by boiling in 4×SDS loading buffer and were separated by SDS-PAGE, followed by Western blotting with anti-GST antibody. **B.** Novobiocin specifically binds to HIF1α CTAD. Protein mixtures of GST-HIF1α CTAD, novobiocin-sepharose and free novobiocin from 0.25 to 8 mM were incubated at 4°C for 2 hours, and the beads were washed with the same ice-cold buffer. Bound proteins were confirmed by Western blotting with anti-GST antibody (upper panel). Quantified proteins are shown in the bar graph (lower panel). P*ercentages* above *each bar* represents bound HIF1α 776 protein compared to lane 2. **C.** HIF1α CTAD and p300 CH1 cannot compete for binding to novobiocin. Pre-equiliblited novobiocin-sepharose (∼20 µl) was mixed with ∼5 µg GST-HIF1α CTAD and Flag-p300 CH1 (5, 10, and 15 µg) in combinations indicated in the figure. Bound proteins were detected by Western blotting with indicated antibodies.

To expand our experiments, we investigated whether novobiocin directly binds to either HIF1α CTAD or p300 CH1. Specifically, we prepared novobiocin-immobilized sepharose. Fig. 4A indicates that sepharose alone pulled down neither HIF1α CTAD nor GST (lanes 1–2). GST-HIF1α CTAD was pulled down by novobiocin-immobilized sepharose (lane 4), but GST was not (lane 3), suggesting that novobiocin might directly bind to HIF1α CTAD. To confirm these results, we performed competitive binding experiments with free novobiocin (0.25–8 mM; Fig. 4B). HIF1α CTAD bound to novobiocin-sepharose was gradually decreased by increasing the amount of free novobiocin. Our data strongly suggest that the inhibitory effects of novobiocin on HIF1α CTAD/p300 CH1 are achieved through direct binding to HIF1α CTAD. To investigate whether novobiocin simultaneously binds to p300 CH1, we performed the same experiments with Flag-p300 CH1. Fig. 4C shows that novobiocin-immobilized sepharose only could only pull down HIF1α CTAD (lane 3), but not p300 CH1 (lane 1). In addition, p300 CH1 could not competitively replace HIF1α CTAD which bound to novobiocin-immobilized sepharose (lanes 4–6).

### Novobiocin-mediated Down-regulation of HIF1α Target Gene CA9 Expression may be Involved in the mTOR Signal Pathway

**Figure 5:**
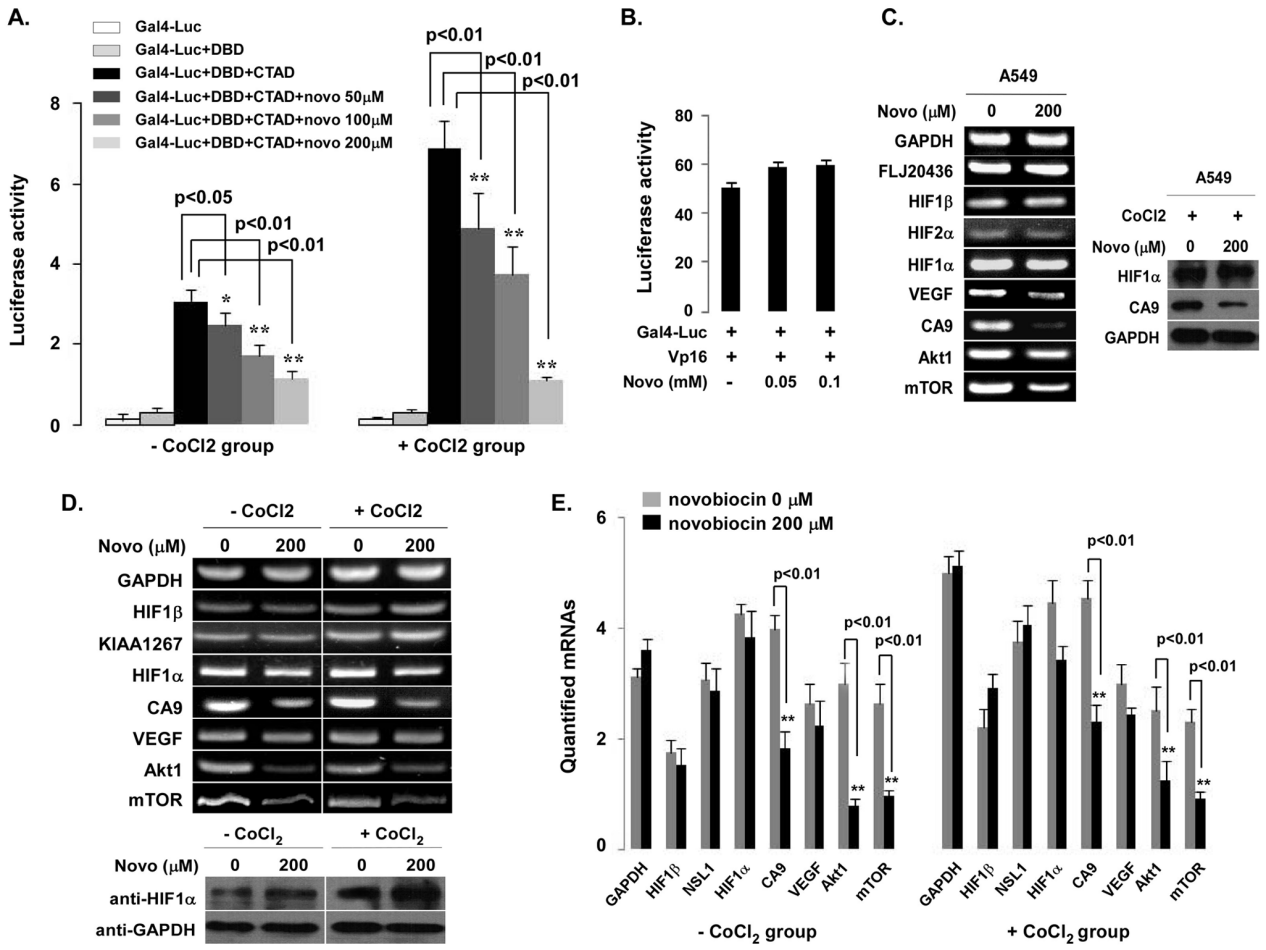
Novobiocin represses the transcriptional activity of HIF1α CTAD and down regulates HIF1α target genes. **A.** Novobiocin inhibits the activation of GAL4-dependent luciferase reporter in 293T cells. 293T cells were co-transfected with luciferase reporter carrying GAL4-Luc and a *Renilla* luciferase plasmid (HIF1α DBD and HIF1α CTAD). After 24 hours transfection, cells were treated with 50, 100, and 200 µM novobiocin with or without CoCl_2_. After 48 *hours* transfection, dual luciferase activities were measured and firefly values were normalized with *Renilla* values. **B.** 293T cells were co-transfected with GAL4-Luc and VP16 plasmid. After 24 hours transfection, cells were treated with 50 and 100 µM novobiocin. **C.** Novobiocin down-regulates HIF1α target genes. A549 cells were cultured with or without 200 µM novobiocin for 24 hours in the presence or absence of CoCl_2_ (150 µM). Selective HIF1α target or non-target gene expressions were measured by RT-PCR (left panel). Western blot analysis was performed with indicated antibodies (right panel). **D.** Novobiocin affects mTOR gene expression in MCF-7 cells. MCF-7 cells were treated with 200 µM novobiocin in the presence or absence of CoCl_2_ (150 µM). Indicated gene expressions including Akt1and mTOR were measured by RT-PCR (upper panel). HIF1α protein is shown in the lower panel. **E.** mRNA was quantified by densitometry using Quantity One software (BioRad) (right panel). Error bars represent the standard error of the mean of 2∼3 independent experiments. The Student’s *t*-test was performed to compare difference between with or without novobiocin treatment (significance: *p<0.05 and **p<0.01).

To verify the effects of novobiocin on HIF1α CTAD-mediated transactivation, we used a Gal4-Luc reporter containing 5-Gal4 binding sites and a HIF1α DNA binding domain (DBD). Co-transfection of HIF1α CTAD in either normal or hypoxic conditions significantly increased luciferase activity, and this activity was inhibited in a dose-dependent by novobiocin (p<0.01; Fig. 5A). However, the Gal4 fusion system with VP16 (Gal4-VP16) was not affected by novobiocin (50–100 µM; Fig. 5B). To further investigate the ability of novobiocin to down-regulate transcription of endogenous HIF1α-inducible genes in cell culture, several cell lines (including A549 and MCF-7) subjected to RT-PCR and relative mRNA expression of selected HIF1α-inducible genes and non-HIF1α-dependent genes were measured. Fig. 5C depicts that novobiocin (200 µM) down-regulated HIF1α-dependent gene expression, including CA9 and VEGF, in both cell lines. In contrast, HIF1α-independent gene expression did not change remarkably (GAPDH, HIF1β and FLJ20436). CA9 is a membrane isoenzyme, the overexpression of which is associated with various human carcinomas such as ovarian, lung, renal, bladder, or breast cancers [26], [27]. Previously, CA9 was reported to promote angiogenesis via enhancing CA9-dependent c-Src/FAK/mTOR activation [28]. To ascertain whether novobiocin-induced down-regulation of CA9 gene expression is associated with the mTOR signal pathway, we evaluated relative mRNA expression of Akt1 and mTOR, key factors in the mTOR signal pathway. We treated cells with novobiocin (200 µM) in the presence or absence of cobalt (150 µM CoCl_2_). Fig. 5C and Fig. 5D show that novobiocin not only significantly reduced CA9 expression with and without cobalt treatment (p<0.01) but it also significantly decreased mRNA expression of AKT1 and mTOR in MCF-7 cells (p<0.01).

### Effects of Novobiocin on Tumor Cell Growth

**Figure 6:**
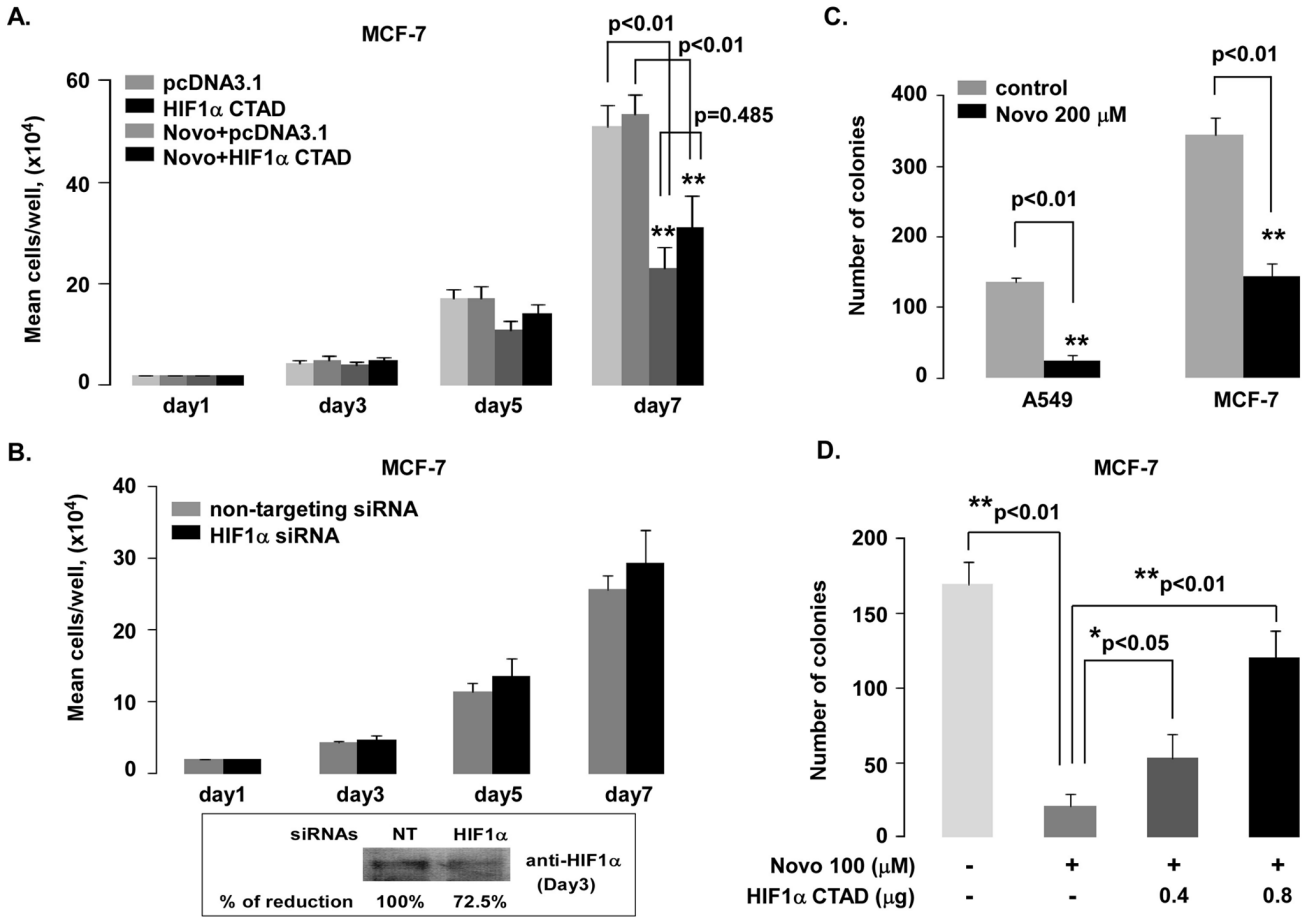
Inhibitory effects of novobiocin on cell proliferation and colony formation in A549 and MCF-7 cells. **A.** Cell growth pattern of MCF-7. MCF-7 cells were transiently transfected with pcDNA3.1 0.8 µg (control) or HIF1α CTAD plasmid 0.8 µg. Later (24 hours), cells were seeded in 12-well plates and grown in medium with (200 µM) or without novobiocin for various days. Cell numbers were counted on a hemacytometer in triplicate each day of the culture, up to the seventh day. **B.** Cell growth numbers were not affected in HIF1α siRNA knockdown MCF-7 cells. MCF-7cells were transfected with 20 nM HIF1α siRNA and non-targeting siRNA (as control) and cultures were treated as described above. As an example, HIF1α protein at Day3 is shown in the bottom panel. **C.** Soft-agar formation assay. Three independent soft agar colony formation assays were performed. Colonies numbers produced by A549 and MCF-7 cells with/without 200 µM novobiocin are shown as bar graphs. **D.** HIF1α CTAD partially rescued the inhibitory effects of novobiocin on colony formation. MCF-7 cells were transiently transfected with pcDNA3.1 plasmid 0.8 µg (control) or HIF1α CTAD plasmid at 0.4 and 0.8 µg. Then (24 hours later), a colony formation assay was performed with or without 100 µM novobiocin. Error bars indicate SE; (significance: *p<0.05 and **p<0.01).

A colony formation assay in soft agar was performed to measure novobiocin’s affects on clonogenic growth. Novobiocin (200 µM) significantly inhibited A549 and MCF-7 colony formation as measured by decreased colony numbers (p<0.01; Fig. 6C). Next, to verify whether the HIF pathway was involved in MCF-7 colony formation, we performed an HIF1α CTAD rescue experiment. Interestingly, a one-time transient transfection of HIF1α CTAD dose-dependently and partially rescued novobiocin-induced reduction of colony formation in MCF-7 cells (Fig. 6D). Colony number data clearly show these rescue effects. Novobiocin (100 µM) almost completely inhibited MCF-7 colony formation (p<0.01 compared to pcDNA3.1 vector-only transfected control group); however, the number of colonies increased in a dose-dependent fashion when transfected with pcDNA3.1/HIF1α CTAD. Compared to novobiocin-treated MCF-7 cells, colony numbers significantly increased with transfection of 0.4 and 0.8 µg pcDNA3.1/HIF1α CTAD plasmids (p<0.05 and p<0.01, respectively). Similar results were observed in cell proliferation assays. In MCF-7 cells, cell growth rates were markedly inhibited in the presence of novobiocin (p<0.01), and this observation was partially recovered by transiently transfecting HIF1α CTAD. No significant differences were observed between novobiocin and HIF1α CTAD transfection groups (p>0.05; Fig. 6A). Although HIF1α protein expression was reduced 72.5% compared to the non-targeting siRNA control group on day 3, cell growth rates were not affected during the 7-day observation period (Fig. 6B).

## Discussion

HIF1α, a potential tumor hypoxic marker, is a key cellular survival protein during hypoxia. Under hypoxic conditions, accumulated HIF1α dimerises with HIF1β and recruits transcriptional co-activators such as p300/CBP and binds to HRE, activating hypoxically regulated genes such as VEGF, GLU-1 and CA9 [1], [4]–[7] CA9 is a transmembrane, zinc-containing metalloenzyme that catalyzes reversible reactions of the bicarbonate buffer system to regulate pH under hypoxic conditions [29], and tissue hypoxia is the main regulator of CA9 expression in tumors [30]. Overexpression of CA9 has been reported in a wide variety of malignant cell lines and tumors, including breast carcinomas, bladder cancer, and lung cancers [31]–[33]. Therefore, CA9 overexpression may be an indicator of early stages of tumor development. Moreover, CA9 has been well-described as a diagnostic marker for clear cell renal carcinoma (ccRCC), and it is highly expressed in metastatic ccRCC (mccRCC) [34]–[36]. Thus, an inhibitor of hypoxic tumor-associated CA9 expression offers therapeutic potential in the treatment of tumors in which CA9 is contributes to the perturbation of the extra- or intra-tumoral acidification process. In our experiments, novobiocin inhibited the expression of selected HIF1α target genes in A549 and MCF-7 cells at the same concentrations, and CA9 expression was more selectively inhibited by novobiocin. These data support the idea that novobiocin and its analogues may be a new class of antitumor drugs which target highly overexpressed-CA9 during tumorigenesis.

Hsp90, a molecular chaperone, is known to be involved in multiple oncogenic pathways and is overexpressed in tumor cells. Many proteins that contribute to cancer cell proliferation and growth are dependent upon Hsp90 for their folding and conformational maintenance [37]. Novobiocin, as a potent inhibitor of bacterial DNA gyrase, binds to the C-terminal of Hsp90 and decreases Hsp90 client proteins in various cancer cell lines [38], [18]. Here, we report that a novel function of novobiocin may be direct binding to the HIF1α C-terminal domain, thereby disrupting the interaction between HIF1α CTAD and the transcriptional co-activator p300 CH1 domain. Disrupting the normal interaction of p300/CBP with HIF1α results in diminished hypoxia-inducible transcription, and down-regulation of HIF1α target genes. Our data show that novobiocin reduced selective HIF1α target gene mRNA, especially that of CA9 in several cancer cell lines. Kim BR *et al.* [28] reported that CA9-transfected cells resulted in elevation of intracellular phosphorylated p-mROR, p-Akt, p-4E-BP1, PI3K, p-FAK and HIF1α protein. In our experiments, except for CA9 expression, novobiocin also decreased mRNA expression of AKT1 and mTOR in both A549 and MCF-7 cells. This suggests a role for novobiocin in the mTOR signaling pathway and indicates a novel molecular mechanism for novobiocin which may lead to new tumor drugs. Luo XG *et al.* reported that novobiocin can dose-dependently inhibit the proliferation and migration of breast cancer cells [17]. In agreement with these findings, our data show that novobiocin not only inhibits the colony formation and cell proliferation of MCF-7 and A549 cells, but also that this inhibition is partially rescued by adding HIF1α CTAD. These results suggest that novobiocin’s ability to inhibit cancer cell proliferation might be partly due to interactions of HIF1α and p300. However, MCF-7 cells were not affected by partial knockdown of HIF1α expression (72.5% of the non-targeting group) with siRNAs in our experimental conditions, suggesting that insufficient knockdown of HIF1α may be insufficient to suppress cell growth. In addition, we cannot exclude the possibility of other mechanisms involved in this rescue process. For example, overexpressed HIF1α CTAD may bind novobiocin in the medium and reduce the effective cellular concentration of novobiocin. If true, the observed growth inhibitory effect might be due to HIF1α-independent mechanisms.

**Figure 7:**
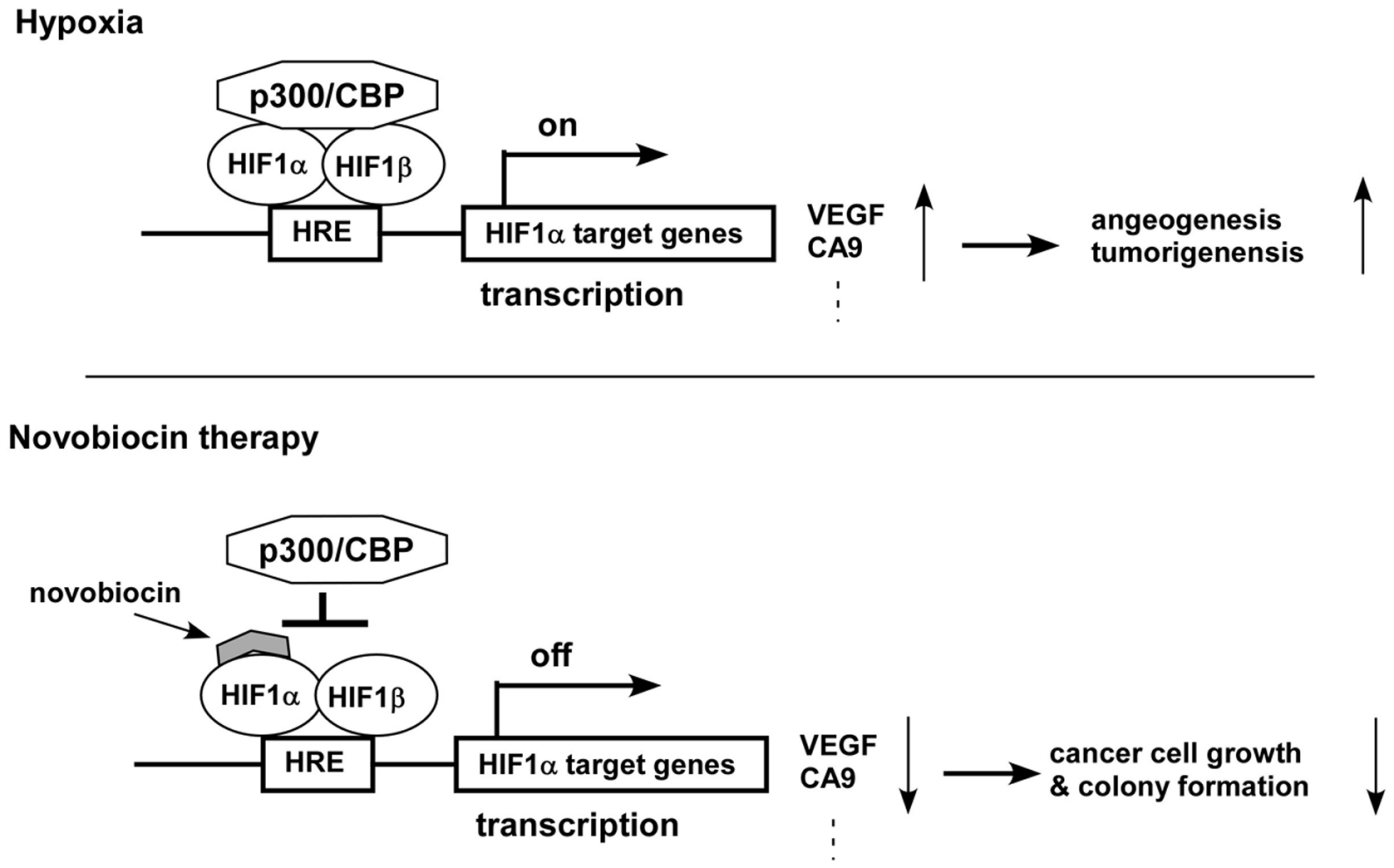
Potential therapeutic model of novobiocin on cancer. Under hypoxia, HIF1**α** dimerises with HIF1β, recruits transcriptional coactivator p300/CBP, and binds to the HRE, thereby leading to activation of hypoxically regulated genes such as VEGF and CA9 (upper panel). Novobiocin directly binds to HIF1α CTAD, inhibits the recruitment of transcriptional co-activator p300/CBP, leading to reduction of hypoxically regulated genes. Ultimately, this suppresses tumor well growth (lower panel).

In summary, our data represent the first report describing the aminocoumarin antibiotic, novobiocin, as a potent inhibitor of bacterial DNA gyrase, which can directly block interaction between the HIF1α CTAD and p300 CH1 complex which down-regulates the transcriptional activation of HIF-responsive genes such as CA9. Also, novobiocin down-regulated mRNA expression of Akt1 and mTOR in both A549 and MCF-7 cells. These data suggest that novobiocin may be involved in the mTOR signaling pathway. In addition, novobiocin dramatically inhibits cell growth and colony formation of human breast adenocarcinoma MCF-7 cells. Rescue experiments indicate that HIF1α CTAD can partially reverse the inhibitory effects of novobiocin on cell growth and colony formation of A549 and MCF-7. Novobiocin-mediated inhibition of cell proliferation in A549 and MCF-7 cells could be partially due to disruptions in the interaction between HIF1α and p300 (Figure 7). Our data suggest that novobiocin may hold promise as a novel chemotherapeutic agent for tumor reduction.
